# Assessing intersectional disparities in obesity among Brazilian adults: a MAIHDA approach

**DOI:** 10.1590/0102-311XEN161425

**Published:** 2026-06-26

**Authors:** Marcos Fanton, Helena Mendes Constante, Raquel Canuto

**Affiliations:** 1 Universidade Federal de Santa Maria, Santa Maria, Brasil.; 2 Department of Sociology, The University of Manchester, Manchester, U.K.; 3 Universidade Federal do Rio Grande do Sul, Porto Alegre, Brasil.

**Keywords:** Obesity, Intersectionality, Multilevel Analysis, Obesidade, Interseccionalidade, Análise Multinível, Obesidad, Interseccionalidad, Análisis Multinivel

## Abstract

Obesity disproportionately affects socially marginalized populations, but traditional analyses often fail to capture the complexity of intersecting social determinants. To address this limitation, we applied the intersectional Multilevel Analysis of Individual Heterogeneity and Discriminatory Accuracy (MAIHDA) to examine how combinations of different systems of power shape obesity prevalence among Brazilian adults. Data from 71,896 individuals from the 2019 *Brazilian National Health Survey* were analyzed. Obesity was defined as body mass index ≥ 30kg/m^2^. Individuals were categorized into 162 intersectional strata based on five dimensions (race, gender, age, income, and education). Two multilevel logistic models were estimated to account for additive and multiplicative effects of these dimensions, and predicted obesity prevalence values across different social strata were examined. The prevalence of obesity varied significantly within and across intersectional strata, with the highest prevalence concentrated among Brown and Black women with lower income and education. The explanatory effect of the intersectional strata decreased from 3.08% to 1.67% after including social dimensions in the model. With a proportional change in variance of 46%, the analysis showed that interaction effects are needed to capture the observed inequities between groups. While additive effects account for part of the variance in obesity, persistent intersectional disparities highlight the limitations of traditional models. These findings underscore the importance of intersectional frameworks in revealing how oppression systems are embodied in health outcomes.

## Resumo

A obesidade afeta desproporcionalmente populações socialmente marginalizadas, mas análises tradicionais frequentemente não conseguem captar a complexidade dos determinantes sociais que a cruzam. Para enfrentar essa limitação, aplicamos a Análise Multinível de Heterogeneidade Individual e Precisão Discriminatória (MAIHDA) interseccional para examinar como combinações de sistemas de poder moldam a prevalência da obesidade em adultos brasileiros. Foram analisados dados de 71.896 indivíduos na *Pesquisa Nacional de Saúde* de 2019. A obesidade foi definida como índice de massa corporal ≥ 30kg/m^2^. Os indivíduos foram categorizados em 162 camadas interseccionais com base em cinco dimensões (raça, gênero, idade, renda e educação). No todo, dois modelos logísticos multinível foram estimados para considerar os efeitos aditivos e multiplicativos dessas dimensões. As prevalências previstas de obesidade em diferentes camadas sociais foram examinadas. A prevalência da obesidade variou significativamente dentro e entre camadas interseccionais, com a maior prevalência concentrando-se em mulheres pardas e negras com menor renda e escolaridade. O efeito explicativo das camadas interseccionais diminuiu de 3,08% para 1,67% após a inclusão das dimensões sociais no modelo. Com uma variância proporcional de 46%, a análise mostrou que os efeitos da interação são necessários para capturar as desigualdades entre os grupos. Embora os efeitos aditivos expliquem parte da variância na obesidade, disparidades interseccionais persistentes destacam as limitações dos modelos tradicionais. Esses achados ressaltam a importância dos quadros interseccionais para revelar como os sistemas de opressão estão incorporados nos resultados de saúde.

## Resumen

La obesidad afecta de manera desproporcionada a las poblaciones socialmente marginadas, pero los análisis tradicionales a menudo no logran captar la complejidad de los determinantes sociales que se entrecruzan. Para abordar esta limitación, aplicamos el Análisis Multinivel de la Heterogeneidad Individual y la Precisión Discriminatoria (MAIHDA) interseccional para examinar cómo las combinaciones de diferentes sistemas de poder configuran la prevalencia de la obesidad en los adultos brasileños. Se analizaron los datos de 71.896 personas de la *Encuesta Nacional de Salud* de Brasil de 2019. La obesidad se definió como un índice de masa corporal ≥ 30kg/m^2^. Las personas se clasificaron en 162 estratos interseccionales basados en cinco dimensiones (raza, género, edad, ingresos y educación). Se estimaron dos modelos logísticos multinivel para tener en cuenta los efectos aditivos y multiplicativos de estas dimensiones, y se examinaron las prevalencias de obesidad previstas en los diferentes estratos sociales. La prevalencia de la obesidad varió significativamente dentro y entre los estratos interseccionales, concentrándose la prevalencia más alta entre las mujeres de los grupos de raza negra y morena con menores ingresos y nivel educativo. El efecto explicativo de los estratos interseccionales disminuyó del 3,08% al 1,67% tras incluir las dimensiones sociales en el modelo. Con un cambio proporcional en la varianza del 46%, el análisis mostró que los efectos de interacción son necesarios para captar las desigualdades observadas entre los grupos. Si bien los efectos aditivos explican parte de la varianza en la obesidad, las persistentes disparidades interseccionales ponen de relieve las limitaciones de los modelos tradicionales. Estos hallazgos subrayan la importancia de los marcos interseccionales para revelar cómo los sistemas de opresión se plasman en los resultados de salud.

## Introduction

Obesity is recognized as a disease of pandemic proportions given its widespread and increasing prevalence worldwide. Projections estimate that by 2050, one in three adults aged over 25 will be living with obesity ^1^. In Brazil, the prevalence of obesity has grown rapidly, affecting 26% of the adult population in 2019 ^2^. This figure is expected to rise to 29.6% by 2030 ^3^. Its health burden is considerable: in 2019, high body mass index (BMI) was responsible for 2,404 disability-adjusted life years (DALYs), comprising 658 years lived with disability (YLDs) and 1,746 years of life lost due to premature death (YLLs), as well as 76 deaths per 100,000 inhabitants in Brazil ^4^.

Obesity is characterized by excessive accumulation of body fat and results from a complex interplay of factors influencing energy balance, including biological, behavioral, psychological, social, political, and commercial determinants ^5^ ^,^ ^6^. Although social determinants of obesity are increasingly recognized in academic and policy discourse, most empirical studies struggle to adequately address these dimensions methodologically. Research on obesity often relies on individual-level frameworks that emphasize behavioral and biomedical risk factors while neglecting the broader structural and systemic drivers that shape population health. As a result, public policies and health interventions tend to reproduce narratives that individualize responsibility for obesity, focusing predominantly on personal choices such as diet and physical inactivity ^7^. This individualistic approach not only overlooks complex social and economic contexts in which obesity develops but also risks reinforcing stigma and blame, particularly among marginalized populations disproportionately affected by the condition.

Obesity does not affect all populations equally. In countries from the Organisation for Economic Co-operation and Development (OECD), research has shown that obesity disproportionately affects socially and economically disadvantaged groups ^8^. In Brazil, similar patterns of inequality have been observed, and previous studies have demonstrated that obesity prevalence is disproportionately higher among women, racial-ethnic minorities, and poorer individuals ^9^.

Intersectionality, as a critical and analytic framework, has increasingly been applied in epidemiological research, particularly in the study of health outcomes ^10^ ^,^ ^11^ ^,^ ^12^.

At its core, intersectionality posits that individuals are situated within complex and interlocking systems of power that shape their lived experiences via mechanisms of oppression or privilege ^13^ ^,^ ^14^ ^,^ ^15^ ^,^ ^16^. Within social epidemiology, intersectionality enables a reconceptualization of individual-level categories (such as gender, race/skin color, income, age, and/or sexuality) as proxies for broader social and structural systems of power, like sexism, classism, ageism, and heteronormativity. The intersection of these categories may give rise to distinct intersectional social strata that reflect cumulative and multiplicative exposures to such systems of power.

Recently, multilevel approaches have enabled researchers to operationalize these theoretical premises in population-level studies. The Multilevel Analysis of Individual Heterogeneity and Discriminatory Accuracy (MAIHDA), introduced by Evans et al. ^17^, incorporates an intersectional lens by nesting individuals (level 1) within more abstract groupings, known as intersectional strata (level 2). Each individual is thereby nested within a unique social position formed by combinations of key social variables (e.g., gender, race, and class) ^17^ ^,^ ^18^.

This study builds on previous research applying MAIHDA to assess intersectional inequalities in obesity and nutrition-related outcomes in other populations ^19^ ^,^ ^20^ ^,^ ^21^ ^,^ ^22^ ^,^ ^23^, demonstrating the relevance of using an intersectional quantitative approach to measure social gradients in health outcomes. In our study, this modeling strategy allowed us to: (1) estimate the prevalence of obesity within each stratum; (2) assess variance between and within strata; and (3) examine the influence of intersecting social identities on obesity rates among Brazilian adults. These objectives are essential to elucidate the role of structural systems of power in shaping obesity ^17^ ^,^ ^24^ ^,^ ^25^ ^,^ ^26^.

## Data and methods

### Data source

This study uses data from the 2019 *Brazilian National Health Survey* (PNS, acronym in Portuguese), a nationwide survey with sampling conducted in three stages: selection of census tracts as primary sampling units (PSUs), selection of households, and selection of residents aged 15 years or older. The questionnaire was divided into household and individual data. The microdata are publicly available at https://ftp.ibge.gov.br/PNS/2019/Microdados/. The survey protocol was approved by the Brazilian National Research Ethics Committee (process n. 3,529,376). Participants were informed about relevant information in accordance with *Resolution n. 466/2012* of the Brazilian National Health Council and provided written informed consent. More information on the survey methodology can be found elsewhere ^27^.

The initial sample comprised 94,114 individuals who completed the interviewer-administered questionnaire. We excluded 17,307 individuals under 18 years of age or over 65 years of age, as well as 738 pregnant women, as these groups may have distinct obesity dynamics or may not accurately reflect adiposity. From this sample, 3,017 individuals had no recorded height or weight, and 52 respondents had implausible BMI values (defined as BMI ≤ 15kg/m^2^ and ≥ 60kg/m^2^) (Supplementary Material - Figure S1; https://cadernos.ensp.fiocruz.br/static//arquivo/suppl-e00161425_5898.pdf) ^28^. Individuals who self-identified as Yellow (n = 523) or Indigenous (n = 552) were excluded due to an insufficient number of observations to construct corresponding intersectional strata. Considering an additional loss of 21 participants without data on household income, our final analytical sample comprised 71,896 individuals with complete data.

### Outcome

Obesity was defined as the outcome of interest. We first calculated BMI as self-reported weight in kilograms divided by the square of self-reported height in meters (kg/m^2^). Individuals with a BMI ≥ 30kg/m^2^ were classified as having obesity ^29^.

### Social strata dimensions

This study selected five sociodemographic variables to construct the social intersectional strata: age, gender, race, income level, and educational level. Age was categorized into tertiles: younger adults (18-34 years), middle-aged adults (35-48 years), and older adults (49-65 years). Gender was assessed via self-reported sex, with response options “male” and “female”, and subsequently classified as “man” and “woman” to reflect binary gender expressions. Self-reported race was assessed using the five categories of the Brazilian Institute of Geography and Statistics: White, Black, Yellow, Brown (“*Pardos*”), and Indigenous, with White, Black, and Brown included in the final analysis. Household per capita income was measured, excluding the income of individuals identified as pensioners, domestic workers, or relatives of domestic workers. This variable was assessed in relation to the minimum wage at the time of the survey (BRL 998.00) and subsequently categorized into “low income” (0-1 minimum wage), “middle income” (1-3 minimum wages), and “high income” (greater than 3 minimum wages). Educational attainment was grouped into “low education” (no schooling to incomplete middle school), “middle education” (complete middle to complete high school), and “high education” (incomplete higher education or above).

All possible combinations of the selected social variables resulted in 162 intersectional strata, with individuals nested within categories of age (three categories), gender (two categories), race (three categories), income (three categories), and education (three categories). Each stratum was labeled using a concatenated string that identified the specific combination of social positions - for example, “Middle-aged Male Brown Low Income Low Education”. Three intersectional strata had no observations: “Younger Women Brown High income Low education”, “Younger Women Black High income Low education”, and “Middle-aged Women Brown High income Low education”.

It is important to stress that the composition of the social strata variable does not imply a biological perspective at any level. Instead, this variable maps individuals’ social location within specific systems of power that shape privilege or oppression across social groups, such as women and men, or White and Black individuals. These categories serve as proxies for social and structural processes, such as racism and patriarchy, enabling interpretations of these systems and their association with obesity and other health disparities ^15^ ^,^ ^16^ ^,^ ^30^. It is also noteworthy that while age has an undeniable biological dimension, it is not solely a biological factor. The experience of aging is shaped by social contexts, giving rise to systems of power that can privilege or oppress individuals based on their age group. For instance, younger individuals may benefit from opportunities denied to older individuals due to ageism, while older adults may experience structural and cumulative discrimination in the workforce or healthcare settings.

### Analytical approach

As a first step in the analytical process, we described relative frequencies of each social dimension according to the overall analytical sample, along with the prevalence of obesity and the corresponding 95% confidence intervals (95%CIs), accounting for the survey’s complex sampling design and sampling weights. We then conducted logistic MAIHDA, given the binary nature of the outcome. Over the past five years, this method has gained increasing prominence and is currently considered a “gold standard” for describing health inequities both between and within intersectional strata across a range of different health outcomes, including BMI ^19^ ^,^ ^20^ ^,^ ^23^. Since its introduction, several limitations and methodological concerns ^31^ have been raised; however, these have been increasingly addressed with clarification and support for its use ^32^.

First, we fitted the unadjusted null model (or variance component model), which serves as a simple intersectional model that estimates the total variance in the outcome between and within all 159 intersectional strata. Second, we specified the full model (or main effects model), incorporating the individual-level social variables used to construct the intersectional strata (age, gender, race, income, and education) as fixed parameters. This model enabled the assessment of the extent to which total variance can be explained by additive or interactive contributions of these social dimensions. For both models, we assigned second-order penalized quasi-likelihood estimates (PQL2) to define initial starting values, and models were subsequently fitted using Markov chain Monte Carlo procedures (MCMC) ^33^. Additionally, we fitted alternative models including only four social variables to define the intersectional strata (age, gender, race, and either income or education); however, these were discarded as they did not improve the main model performance indicators (Supplementary Material; https://cadernos.ensp.fiocruz.br/static//arquivo/suppl-e00161425_5898.pdf).

We also specified three key measures to understand the relationship between intersectional strata and the outcome, which is one of the main goals of the MAIHDA approach ^18^. The variance partition coefficient (VPC) was the first measure of discriminatory accuracy considered. In the null model, the VPC indicates the proportion of total variance in obesity attributable to differences between intersectional strata. In the full model, while the VPC retains the same interpretation, it also reflects the extent to which additive effects of social dimensions included in the intersectional strata explain variation between strata. Theoretically, a VPC close to zero in the full model would suggest that inequities in obesity are predominantly additive (i.e., driven by each social dimension rather than by interactions among these dimensions). Thus, the VPC serves as a global measure to assess whether the MAIHDA framework is necessary or whether traditional regression methods would suffice to estimate marginal effects and predict inequities in this sample. The proportional change in variance (PCV) was also assessed, as it measures the extent to which between-strata variance is explained while controlling for the additive main effects included in the full model. A PCV close to 100% suggests that the additive social dimensions fully account for the variance between strata, thereby minimizing interaction effects. Finally, we examined the area under the receiver operating characteristic curve (AUC), which reflects the probability that an individual with obesity belongs to a stratum with a higher predicted prevalence of obesity than an individual without obesity. The AUC ranges from 0.5 (no discriminatory ability) to 1.0 (perfect discrimination) ^18^.

All analyses were performed using Stata 18.0 (https://www.stata.com), and MAIHDA models were fitted using MLwiN 3.07 software (http://www.bristol.ac.uk/cmm/software/mlwin/) within Stata using the *runmlwin* function. The do-files used in this study will be available at https://github.com/marcosfanton/maihda_csp.

## Results

The analytical sample was equally distributed across age groups (Table 1), with a mean age of 40.44 years (standard deviation - SD = 13.32). Slightly over half of the sample consisted of women (52.06%), and participants predominantly self-identified as Brown (45.57%) or Black (11.83%), had lower income (52.49%), and had low or middle levels of educational attainment (77.54%). The overall prevalence of obesity was 21.59% (95%CI: 20.80; 22.40), with higher prevalence values observed among middle-aged and older adults, women, Brown and Black individuals, and those with lower levels of education.

**Table 1:** Distribution of the analytical sample and prevalence of obesity (body mass index - BMI ≥ 30kg/m^2^) according to social dimension strata. *Brazilian National Health Survey*, 2019.

Variables	Sample distribution		Obesity
	n	% (95%CI)	% (95%CI)
Age groups			
Younger adults	26,278	36.55 (35.85; 37.26)	16.13 (15.11; 17.21)
Middle-aged adults	22,964	31.94 (31.32; 32.57)	25.16 (23.75; 26.64)
Older adults	22,654	31.51 (30.92; 32.10)	24.28 (23.34; 25.25)
Gender			
Men	34,467	47.94 (47.28; 48.59)	19.81 (18.84; 20.82)
Women	37,429	52.06 (51.41; 52.72)	23.22 (22.33; 24.13)
Race			
White	30,628	42.60 (41.82; 43.39)	21.11 (20.00; 22.26)
Brown	32,763	45.57 (44.84; 46.30)	21.42 (20.52; 22.34)
Black	8,505	11.83 (11.39; 12.29)	23.96 (22.40; 25.59)
Minimum wage *			
Low (≤ 1)	37,738	52.49 (51.62; 53.36)	21.24 (20.31; 22.19)
Middle (1-3)	26,350	36.65 (35.91; 37.40)	22.58 (21.39; 23.81)
High (> 3)	7,808	10.86 (10.25; 11.49)	19.91 (18.47; 21.44)
Educational level			
Low	21,245	29.55 (28.87; 30.24)	23.48 (22.52; 24.47)
Middle	34,503	47.99 (47.27; 48.71)	21.41 (20.19; 22.69)
High	16,148	22.46 (21.68; 23.27)	19.46 (18.40; 20.57)
Total	71,896	100.00	21.59 (20.80; 22.40)

95%CI: 95% confidence interval.

Note: all estimates (relative frequencies, obesity prevalence, and 95%CI) were calculated considering the complex sampling design and sampling weights.

* In 2019, the minimum wage in Brazil was BRL 998,00.

Results from both MAIHDA logistic models (null and main effects models) are presented in Table 2. In the null model, the VPC for obesity was estimated at 3.08%, suggesting that 3% of the total variance in obesity prevalence is attributable to differences between intersectional strata. When the social dimensions used to define the intersectional strata were added as fixed effects (full model), the VPC declined to 1.65%. Although the reduction in VPC indicates that part of the between-stratum variance in obesity prevalence is explained by the additive main effects of these social dimensions, the persistence of a non-negligible VPC in the full model suggests that intersectional (non-additive) effects remain. This interpretation is supported by the PCV, which was estimated at 46.42%. The fixed-effect regression coefficients from the full model (Table 2) represent odds ratios (ORs) from the additive component of the model. Although these are not the primary focus of MAIHDA, they help illustrate the additive (non-interactive) and unilateral influence of broader social dimensions on obesity prevalence. For example, compared to younger adults, middle-aged individuals (OR = 1.56, 95%CI: 1.41; 1.73) and older adults (OR = 1.53, 95%CI: 1.35; 1.69) had significantly higher odds of obesity. Black individuals (OR = 1.15, 95%CI: 1.02; 1.29) also had higher odds compared to white individuals, while no significant differences were observed for Brown individuals. Regarding gender, educational attainment, and income levels, no clear pattern emerged.

**Table 2:** Parameter estimates from multilevel logistic regression models of obesity (body mass index - BMI ≥ 30kg/m^2^) in the analytical sample (n = 71,896) from the 2019 *Brazilian National Health Survey*.

Parameters	Null model	Full model
	OR (95%CI)	OR (95%CI)
Fixed-effect parameters		
Age groups		
Younger adults		Reference
Middle-aged adults		1.56 (1.41; 1.73)
Older adults		1.53 (1.35; 1.69)
Gender		
Men		Reference
Women		1.11 (1.00; 1.21)
Race		
White		Reference
Brown		1.01 (0.91; 1.12)
Black		1.15 (1.02; 1.29)
Minimum wage *		
Low (≤ 1)		Reference
Middle (1-3)		1.07 (0.96; 1.20)
High (> 3)		0.98 (0.85; 1.11)
Educational level		
Low		Reference
Middle		1.00 (0.90; 1.13)
High		0.90 (0.79; 1.02)
Model intercept (β_0_)	0.27 (0.26; 0.29)	0.18 (0.16; 0.22)
Random-effects parameters		
Between-strata variance (SD)	0.10 (0.08; 0.14)	0.05 (0.04; 0.08)
Summary statistics		
VPC (%)	3.08	1.65
PCV (%)		47.02
AUC	0.59	0.59
Bayesian DIC	72,958.59	72,951.10

95%CI: 95% confidence interval; AUC: area under the receiver operating characteristic curve; DIC: deviance information criterion; OR: odds ratio; PCV: proportional change in variance; VPC: variance partition coefficient.

**Figure 1:**
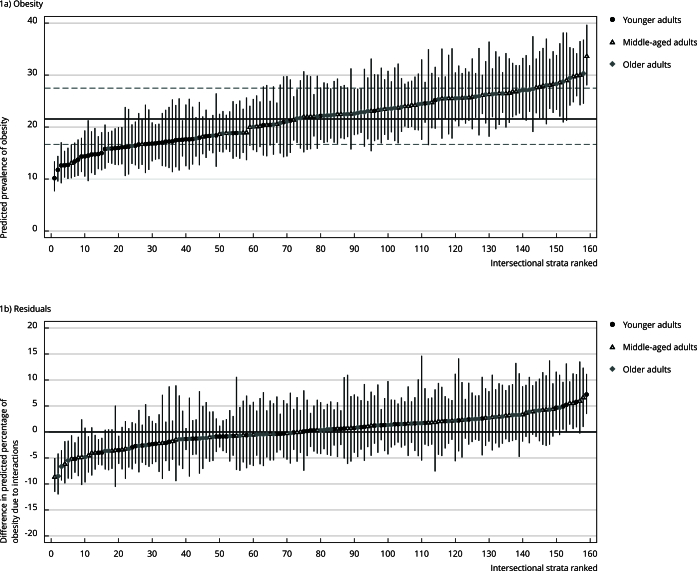
Predicted prevalence of obesity (body mass index - BMI ≥ 30kg/m^2^) and random-effects residuals of the full model across intersectional strata in the analytical sample (n = 71,896) from the 2019 *Brazilian National Health Survey*.

The predicted probabilities of obesity across intersectional strata following the full model are presented in Figure 1a, with highlights shown in Table 3. The lowest predicted prevalence of obesity was observed in “Younger Women White High income High education” (10.18%, 95%CI: 7.66-13.42), and the highest in “Middle Women Brown Low income Low education” (33.48%, 95%CI: 27.55; 39.97). Figure 1b shows that both groups had values that were statistically significantly different from the overall predicted prevalence (21.55%, 95%CI: 16.65; 27.48). The five strata with the lowest predicted prevalence of obesity (ranging from 10.18% to 12.72%) predominantly consist of younger individuals with high levels of education, particularly women. However, younger Black men with low income and lower educational levels are also among these five lowest-prevalence strata. Conversely, the five strata with the highest predicted prevalence (ranging from 29.63% to 33.48%) consisted exclusively of women who were middle-aged or older, from Brown and Black groups, all of whom had low or middle income and educational levels.

**Table 3:** Description of intersectional strata according to predicted obesity prevalence and residuals based on the full model.

Strata	Obesity		Position in Figure 1a
	%	95%CI	
Five lowest-ranked strata of predicted obesity prevalence			
Younger Women White High income High education	10.18	7.66; 13.42	1
Younger Men Black Low income Mid education	11.72	9.69; 14.11	2
Younger Men Black Low income Low education	12.58	10.32; 15.26	3
Younger Women White Mid income High education	12.65	10.01; 15.86	4
Younger Women Black High income High education	12.72	9.21; 17.32	5
Five highest-ranked strata of predicted obesity prevalence			
Middle Women Brown Low income Mid education	29.63	24.25; 35.64	155
Middle Women Black Mid income Low education	29.74	23.70; 36.58	156
Middle Women Black Low income Low education	29.86	25.83; 34.22	157
Older Women Brown Low income Mid education	30.41	24.29; 37.31	158
Middle Women Brown Low income Low education	33.48	27.55; 39.97	159
Below the lowest predicted 95%CI (lower dashed line in Figure 1a - 16.65)			
Younger Women White High income High education	10.19	7.66; 13.43	1
Younger Men Black Low income Mid education	11.73	9.70; 14.11	2
Younger Men Black Low income Low education	12.59	10.32; 15.26	3
Younger Women White Mid income High education	12.65	10.01; 15.86	4
Middle Women White High income High education	13.00	10.21; 16.42	6
Above the highest predicted 95%CI (higher dashed line in Figure 1a - 27.48)			
Middle Women Brown Low income Low education	33.48	27.55; 39.97	159
Strata	Residuals		Position in Figure 1b
	%	95%CI	
Below the reference line (zero)			
Middle Women White High income High education	-8.85	-11.60; -5.36	1
Older Men Brown Low income Low education	-7.98	-11.24; -4.10	2
Older Men Black Low income Low education	-6.27	-8.76; -3.44	3
Middle Men Brown Low income Low education	-5.81	-9.67; -1.14	4
Middle Women Black High income High education	-5.13	-9.07; -0.28	5
Younger Women White High income High education	-5.08	-7.74; -1.90	6
Older Men Black Mid income Low education	-4.99	-8.31; -1.24	8
Middle Women White Mid income High education	-4.94	-8.51; -0.96	9
Middle Men White Low income Low education	-4.27	-7.36; -0.71	10
Younger Men Black Low income Mid education	-3.99	-6.11; -1.62	11
Younger Women White Mid income High education	-3.87	-6.27; -1.12	13
Middle Men Black Low income Low education	-3.67	-6.42; -0.64	16
Younger Men Black Low income Low education	-3.11	-5.47; -0.55	22
Above the reference line (zero)			
Younger Women White Low income Low education	5.02	0.73; 9.58	152
Middle Women Black Low income Low education	5.55	1.43; 9.98	153
Middle Men Black Mid income High education	5.67	1.18; 11.19	154
Middle Men White High income Mid education	5.85	0.09; 12.86	156
Younger Women Brown Low income Low education	6.39	1.18; 13.45	157
Middle Women Brown Low income Low education	6.72	0.74; 13.78	158
Younger Women Black Low income Low education	7.31	3.39;11.45	159

95%CI: 95% confidence interval.

## Discussion

This study aimed to analyze the extent to which different and mutually reinforcing systems of oppression and privilege (age, gender, race, class, and education) shape unequal population-level patterns of obesity and the magnitude of inequality across social strata in a representative sample of Brazilian adults. By implementing MAIHDA within an intersectional framework, we estimated obesity prevalence across intersectional social positions and calculated global measures to assess the contribution of interactive social effects. Our findings reveal stark intersectional inequalities in obesity prevalence, with the predicted prevalence among middle-aged Brown women with low income and low educational attainment three times higher than that among younger White women with high income and high education. Additionally, obesity prevalence varied significantly within and across intersectional strata, with the highest prevalence concentrated among Brown and Black women with lower income and educational levels.

Since the 2010s, studies have increasingly adopted broader theoretical frameworks from social epidemiology to understand trends in malnutrition, including political and social determinants of health ^34^ ^,^ ^35^ ^,^ ^36^ ^,^ ^37^ ^,^ ^38^. These frameworks consider not only individuals’ socioeconomic position but also the structure and dynamics of food systems, such as food security, presence of food deserts, public policies, and the implementation of the human right to adequate food ^6^. From this perspective, structural and systemic factors provide greater explanatory power than individual choices, even if such relationships are more indirect and interactive. This aligns with a growing consensus that rejects biological determinism and individualistic or culturally stereotyped explanations for disparities in obesity rates ^39^. Instead, ecosocial and social epidemiological theories emphasize that health inequalities reflect how individuals and social groups embody intersecting systems of privilege and oppression over the life course ^36^ ^,^ ^37^ ^,^ ^38^ ^,^ ^40^. These theoretical frameworks gain further empirical support in the Brazilian context, where evidence demonstrates how structural inequalities shape obesity patterns across intersecting social markers. Findings from both Canella et al. ^9^ and Araujo et al. ^41^, based on data from the 2008-2009 *Brazilian Household Budget Survey*, provide consistent evidence of associations between socioeconomic positions (such as age, gender, race/ethnicity, income, and education) and overall higher obesity rates. Araujo et al. ^41^ highlight that the association between obesity and race among Brazilian adults is significantly modified by sex and socioeconomic status. The authors reported that obesity prevalence was highest among Black women (20.6%) and lowest among Brown men (11.8%). Among men, obesity increased with higher socioeconomic status across all racial groups, with Black men showing the steepest gradient. Among women, however, white and Brown women showed decreasing odds of obesity with increasing socioeconomic status, whereas Black women exhibited the opposite pattern, presenting three times greater odds of obesity at higher socioeconomic status levels compared to their low-socioeconomic status counterparts. Canella et al. ^9^ observed a high overall prevalence of overweight and obesity among adult women with lower income and educational attainment and among those who self-identified as Black or Brown, compared with women from privileged social positions.

In our study, we assessed the influence of intersectional strata on obesity prevalence by considering each variable as distinct axes of power that are structurally and systematically embedded within social and political institutions, as articulated by Crenshaw ^15^, Collins ^13^, and Collins & Bilge ^30^, among others. Our MAIHDA models indicate that differences between intersectional social strata exert a modest influence on disparities in obesity prevalence. The results initially showed that only 3.08% of the total variance in predicted obesity prevalence is attributable to differences between intersectional strata defined by gender, race, class, education, and age. As emphasized in the literature, the VPC captures both between-strata variability and residual heterogeneity within strata. Values below 5% suggest that most of the observed variation in obesity occurs among individuals within the same stratum rather than between strata ^32^. However, while a low VPC suggests limited discriminatory accuracy of intersectional strata in predicting individual-level outcomes, it does not preclude the presence of meaningful interaction effects between social positions. Recent scholarship demonstrates that even modest between-strata variance can coexist with substantively important within-strata heterogeneity, especially when social categories intersect in non-additive and structurally embedded ways ^18^ ^,^ ^42^. From this perspective, MAIHDA models are not primarily intended to maximize predictive accuracy but rather to reveal patterns of social inequality emerging from intersecting systems of power and privilege ^17^. Consequently, low VPC values should not be interpreted as evidence against the relevance of intersectional effects; instead, they reflect the complex and diffuse ways in which structural determinants operate across and within social groups. In practical terms, these results suggest that these social categories are not precise predictors of individual risk (i.e., with high discriminatory accuracy). Nonetheless, at the population level, they still reveal unequal patterns in the distribution of obesity across social groups ^18^ ^,^ ^43^.

When focusing on the five intersectional strata with the highest predicted prevalence of obesity, a salient pattern emerges. Afro-Brazilian women are the only group consistently represented among these strata, especially those aged 35-48 years with low or intermediate income and educational attainment. The two strata with the highest obesity prevalence include middle-aged and older Brown and Black women with both low income and lower to middle levels of education. Conversely, the five strata with the lowest predicted obesity prevalence are less homogeneous. They predominantly include younger women with varying income levels but consistently high levels of education. Younger Black men with low income and middle to lower educational levels also appear among these groups. While no single intersectional group predominates, these strata share characteristics associated with younger age positioning. These last findings demonstrate how systems of power may generate similar patterns while being driven by entirely different mechanisms and dynamics, such as the formation of structures of oppression or, conversely, of privilege.

These disparities may be partially explained by the chronic activation of stress-related physiological systems in response to intersecting forms of discrimination, including racism, sexism, and classism. As shown in prior research ^44^, experiences of social exclusion and discrimination can activate the hypothalamic-pituitary-adrenal (HPA) axis, increasing levels of cortisol and related hormones. This biological response has been associated with increased visceral fat accumulation, dysregulation of appetite-related hormones, and a greater propensity to consume calorie-dense and highly palatable foods. From an ecosocial perspective, these processes reflect the embodiment of oppression, whereby adverse social conditions are biologically internalized and contribute to health disparities ^35^ ^,^ ^37^ ^,^ ^38^. At a structural level, institutional racism and other political determinants of health shape food environments and access to adequate nutrition ^45^. Discriminatory policies and unequal urban planning contribute to the creation of food deserts and limit the availability of fresh and minimally processed foods in predominantly Black and low-income neighborhoods ^46^ ^,^ ^47^ ^,^ ^48^. Additionally, targeted marketing of ultra-processed foods and the erosion of traditional food practices reinforce unhealthy dietary patterns. Rather than being random or isolated, these patterns reflect historically produced and structurally maintained inequities in living conditions, access to resources, and exposure to stressors. This intersectional analysis underscores the importance of moving beyond additive explanations to identify the compounding effects of systemic social oppression on health outcomes.

### Strengths and limitations

This study presents four main strengths. First, it draws on data from the 2019 PNS, a large and nationally representative sample of Brazilian adults, enabling national-level estimates of obesity prevalence across diverse social strata. Second, the analysis incorporates sampling weights and accounts for the survey’s complex design, thereby increasing validity and generalizability of the results. Third, the implementation of the MAIHDA approach represents a significant methodological and theoretical innovation in Brazilian nutritional epidemiology. This model enables the estimation of prevalence values across all intersectional social strata, capturing both additive and interactive effects and quantifying heterogeneity within and between strata. Moreover, because multilevel models apply shrinkage based on stratum sample size and variance components, MAIHDA provides more reliable estimates for small or sparsely populated strata. Hence, it advances previous studies based on single-level models, which depend on a restricted number of pre-specified interactions and reference groups to remain interpretable and often encourage single-axis interpretations by modeling only additive effects ^24^. By adopting an intersectional perspective, MAIHDA also enables the reconceptualization of traditionally individual or biological variables as markers of social position, thereby expanding the scope of social and ecosocial epidemiological research in nationwide Brazilian studies. Fourth, the study highlights specific high-risk groups (e.g., low-income, middle-aged Black and Brown women), offering actionable insights for targeted public health interventions and equity-oriented policies.

Nonetheless, the study has some limitations. The intersectional strata were constructed using self-reported categorical variables (age, sex, race, income, and education) as proxies for larger structural forces, which may fail to fully capture the complexity of interwoven systemic oppressions such as sexism or racism. Second, despite the large sample size, small cell counts in some strata limited statistical power and required the exclusion of Indigenous and Yellow individuals, thereby reducing inclusivity and obscuring inequalities experienced by these populations. Third, gender was inferred from binary sex categories, restricting the capacity to represent non-binary and transgender individuals, whose interactions with health and social systems may differ substantially. Sexual orientation could also not be included due to insufficient sample size from the PNS 2019 thematic model. Fourth, obesity was estimated using BMI, which is subject to misclassification bias and does not capture body composition, fat distribution, or sociocultural meanings of body size. Finally, while the study adopts an intersectional and ecosocial perspective, the models do not directly incorporate meso- or macro-level variables (e.g., neighborhood food environments, regional policy differences), which could help elucidate structural pathways linking social position to obesity. Future studies should seek to address these limitations when possible.

Taken together, the study provides an important methodological and theoretical contribution to nutritional epidemiology in Brazil, highlighting how structural inequalities are embodied at the intersection of race, gender, age, income, and education. Despite the modest discriminatory accuracy of intersectional strata, our findings reveal clear patterns of vulnerability, with Black and Brown women from lower socioeconomic backgrounds consistently facing higher predicted obesity rates. These results reinforce the limitations of traditional analytical approaches and underscore the need to adopt intersectional and ecosocial frameworks.

Policymakers should prioritize intersectional strategies that address the upstream social and structural determinants of obesity, including racialized poverty, food deserts, and structural barriers to accessing healthy foods in marginalized communities. In the context of Brazil’s ongoing nutrition transition, marked by increasing consumption of ultra-processed foods, it is also essential to monitor social inequalities in obesity with intersectional approaches.

## Data Availability

The databases used in the study, including extraction codes, analyses, and results, are available in the repository: https://github.com/marcosfanton/maihda_csp.
