# Supplementary material for: Assessing intersectional disparities in obesity among Brazilian adults: a MAIHDA approach
Source: Cad Saude Publica. 2026 Jun 26;42:e00161425. doi: 10.1590/0102-311XEN161425 (PMC13313154; doi:10.1590/0102-311XEN161425)
Supplement: Supplementary Material [file 1678-4464-csp-42-EN161425-s.pdf]

SUPPLEMENTARY MATERIAL

Figure S1 Relationship between BMI (kg/m^2) and age in years. Brazilian National Health Survey, 2019.

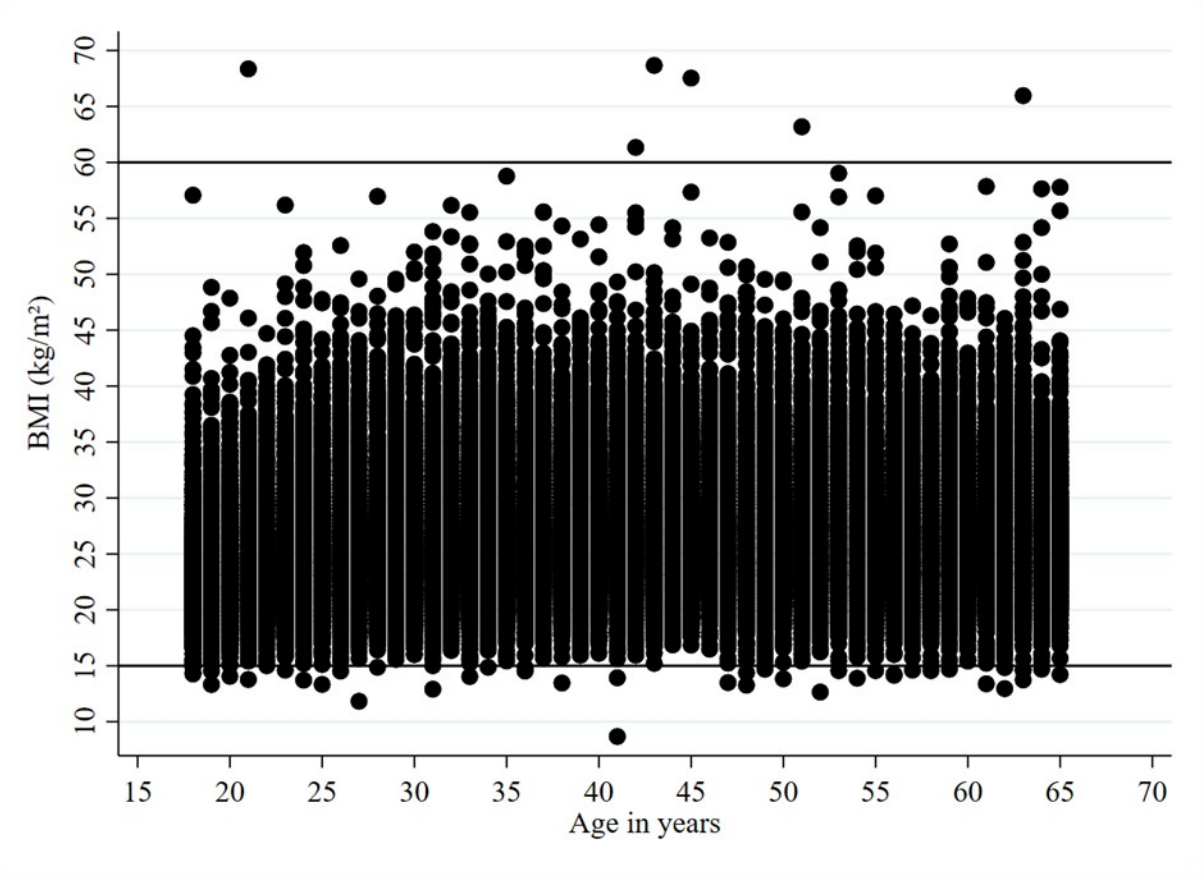

Figure S2 Predicted prevalence of obesity (BMI≥30kg/m2) of the full model across intersectional strata (age, gender, race, income) considering an analytical sample (n=71,896) from the 2019 Brazilian National Health Survey.

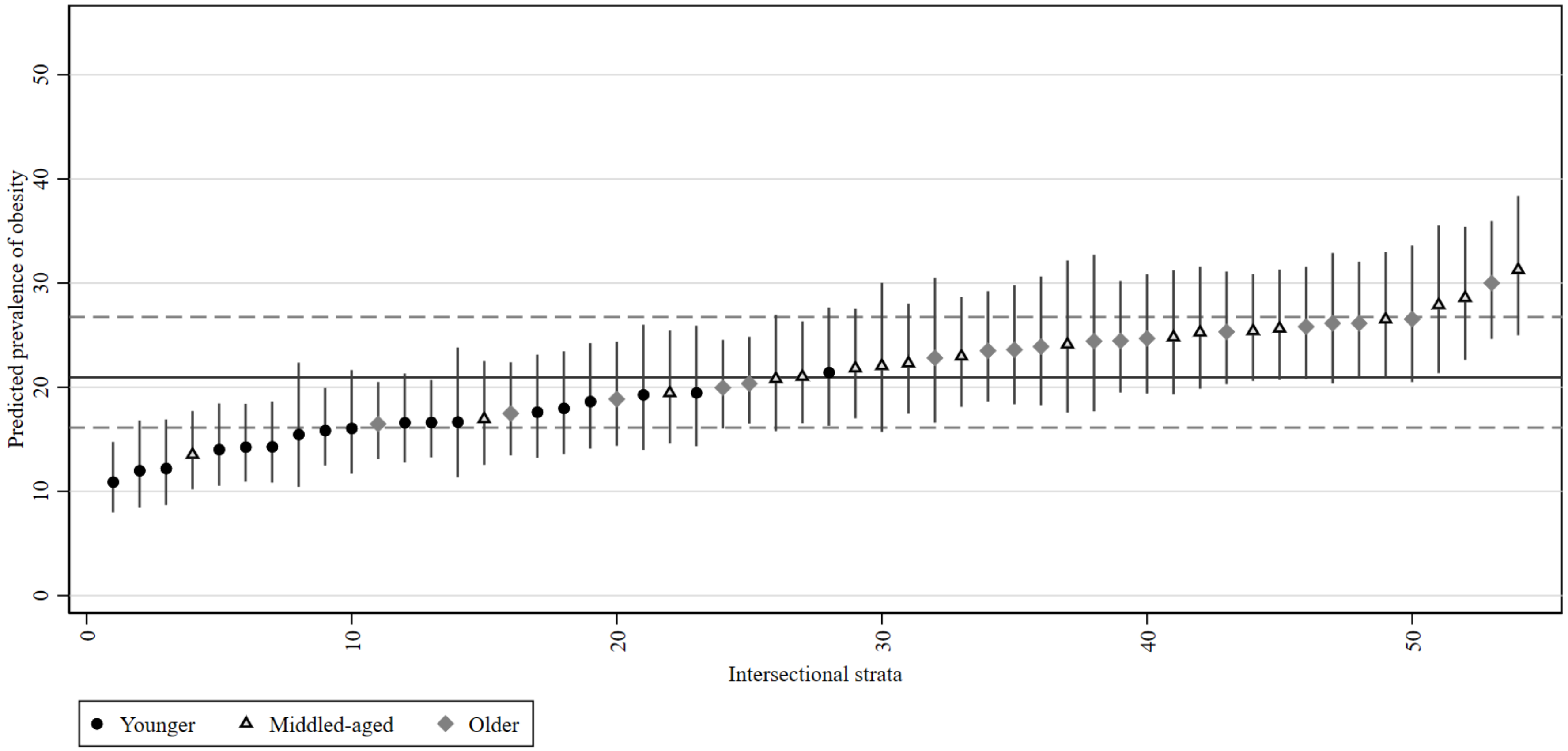

Table S1 Description of the intersectional strata of the predicted prevalence of obesity considering the intersections of age, gender, race, and income.

Rank	Strata	Obesity		Position in Suppl Fig 2
		%	95% CI	
**Five Lowest ranked strata of predicted prevalence of obesity**				
	Younger Women White High income	10.92	7.99-14.75	1
	Younger Women Black High income	12.01	8.44-16.82	2
	Younger Men Black Low income	12.22	8.70-16.91	3
	Middle-aged Women White High income	13.53	10.21-17.73	4
	Younger Men White High income	14.04	10.55-18.44	5
**Five Highest ranked strata of predicted prevalence of obesity**				
	Older Women White Mid income	26.54	20.50-33.61	50
	Middle-aged Women Black Low income	27.90	21.36-35.54	51
	Middle-aged Women Brown Mid income	28.59	22.62-35.40	52
	Older Women Brown Low income	30.00	24.64-35.98	53
	Middle-aged Women Brown Low income	31.28	24.99-38.35	54
**Below the lowest predicted 95% CI**				
	Younger White Women High income	10.91	7.98-14.75	1
**Above the highest predicted 95% CI**				
	-			

Figure S3 Predicted prevalence of obesity (BMI≥30kg/m2) of the full model across intersectional strata (age, gender, race, education) considering an analytical sample (n=71,917) from the 2019 Brazilian National Health Survey.

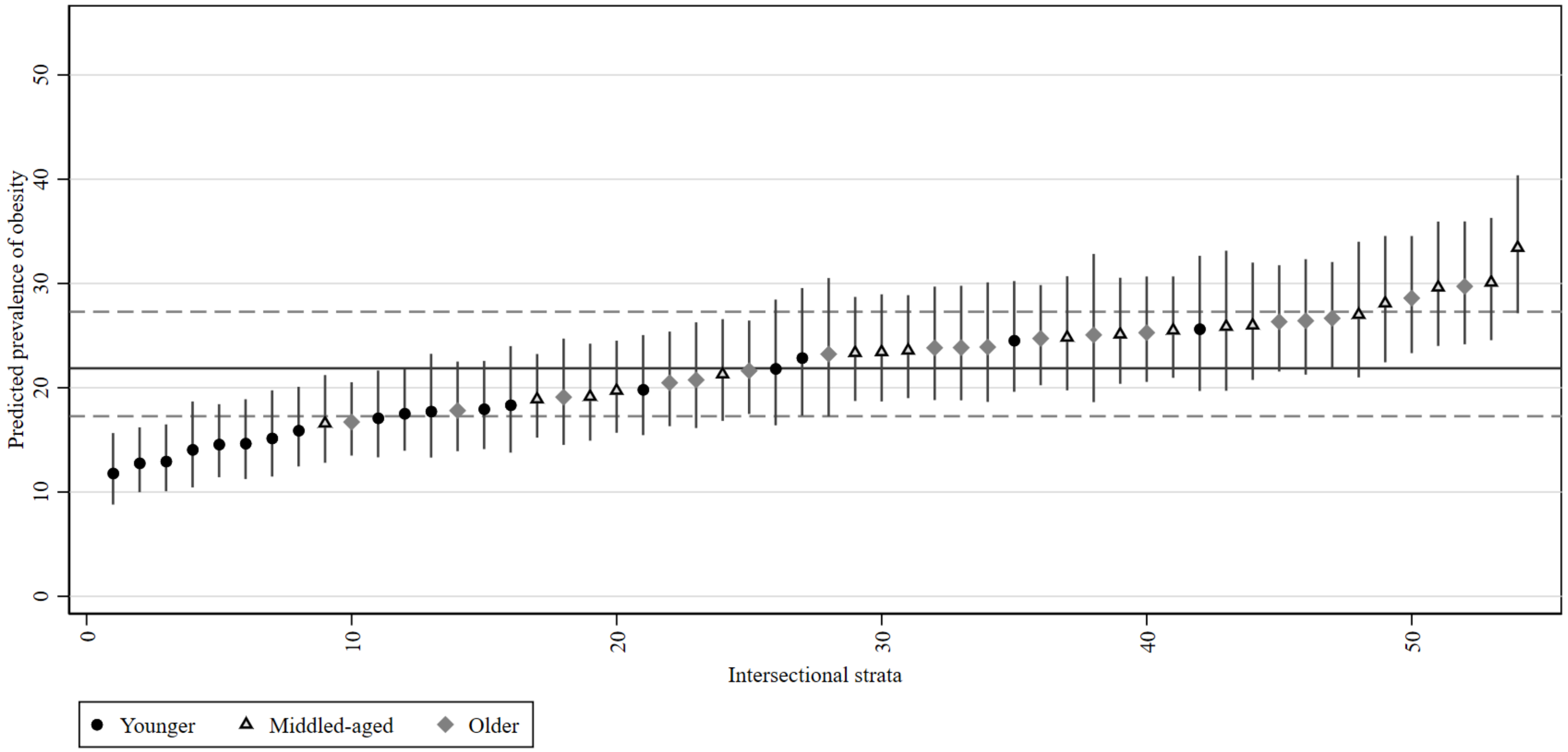

Table S2 Description of the intersectional strata of the predicted prevalence of obesity considering the intersections of age, gender, race, and education.

Rank	Strata	Obesity		Position in Suppl Fig 3
		%	95% CI	
**Five Lowest ranked strata of predicted prevalence of obesity**				
	Younger Women White High education	11.80	8.80-15.66	1
	Younger Men Black Low education	12.78	9.99-16.20	2
	Younger Men Black Mid education	12.95	10.08-16.49	3
	Younger Men White Low education	14.07	10.44-18.68	4
	Younger Women Black High education	14.58	11.42-18.42	5
**Five Highest ranked strata of predicted prevalence of obesity**				
	Older Women Brown Low education	28.60	23.32-34.55	50
	Middle-aged Women Brown Mid education	29.63	24.01-35.94	51
	Older Women Brown Mid education	29.73	24.17-35.95	52
	Middle-aged Women Black Low education	30.10	24.56-36.29	53
	Middle-aged Women Brown Low education	33.44	27.14-40.38	54
**Below the lowest predicted 95% CI**				
	Younger Women White High education	11.80	8.80-15.66	1
	Younger Men Black Low education	12.78	9.99-16.20	2
	Younger Men Black Mid education	12.95	10.08-16.49	3
**Above the highest predicted 95% CI**				
	-			
